# Alzheimer’s Disease-Like Brain Pattern Biomarker: Capturing Risks and Predicting Disease Onset

**DOI:** 10.21203/rs.3.rs-6770628/v1

**Published:** 2025-09-15

**Authors:** Peter Kochunov, Si Gao, Lauren Salminen, Neda Jahanshad, Talia Nir, Paul Thompson, Xiaoming Du, Bhim Adhikari, Alice Kochunov, Ryan Cassidy, Yizhou Ma, Joshua Chiappelli, Seth Ament, Yezhi Pan, Shuo Chen, Alan Shuldiner, Braxton Mitchell, Jair Soares, Elliot Hong

**Affiliations:** The University of Texas Health Science Center at Houston; The University of Texas Health Science Center at Houston; University of Southern California; University of Southern California; University of Southern California Keck School of Medicine; Imaging Genetics Center, Mark and Mary Stevens Neuroimaging & Informatics Institute, Keck School of Medicine of the University of Southern California, Marina del Rey, California, USA; University of Maryland Baltimore; Maryland Psychiatric Research Center; Maryland Psychiatric Research Center; The University of Texas Health Science Center at Houston; The University of Texas Health Science Center at Houston; University of Maryland Baltimore; University of Maryland School of Medicine; University of Maryland Baltimore; University of Maryland School of Medicine; University of Maryland School of Medicine; University of Maryland School of Medicine; McGovern Medical School

**Keywords:** Big data, Alzheimer’s disease, neuroimaging, RVI, amyloid, cerebrovascular disease

## Abstract

Preventing Alzheimer’s disease (AD) requires early-warning biomarkers. We developed a Regional Vulnerability Index (RVI) that quantifies individual brain similarity to AD patients' expected brain deficit patterns. We calculated regional effect sizes to establish brain deficit patterns in amyloid-positive AD cases compared to amyloid-negative healthy controls. The RVI-AD was calculated as a linear index of individual similarity to this established brain pattern in AD. Initially, we demonstrated AD elevation associated with risk factors in 335 participants (mean age: 49±13 years) in the Amish Connectome Project, followed by an independent sample consisting of 26,010 participants (mean age: 64±7 years) from the UK Biobank. Genetic and cardiovascular risks were evaluated using APOE-e4 genotype and Framingham Cardiovascular Risk Scores (FCVRS), respectively. Additionally, we assessed the risk of converting from MCI to dementia in N=1,932 participants (mean age: ~74) from the Alzheimer’s Disease Neuroimaging Initiative (ADNI). Participants with the APOE-e4 allele had significantly elevated RVI-AD indices (p<0.05); FCVRS significantly contributed to higher RVI-AD in an APOE-e4-specific manner (p<0.01), replicable across the samples. In the ADNI cohort, RVI-AD significantly predicted conversion from MCI to dementia in the next decade, particularly within the first 3 years (AUC=74%). In healthy individuals, the RVI-AD index detected the insidious impact of APOE-ε4 and cardiovascular risks in otherwise normally aging cohorts. Elevated RVI-AD also predicted conversion to dementia within 10 years in the older, high-risk cohort. Further development of this brain-pattern similarity-based approach may yield a noninvasive, clinically accessible biomarker to aid early detection of the subtle to more imminent effects of AD risks.

## Introduction

Alzheimer’s disease (AD) is the leading cause of cognitive decline and functional disability among people over the age of 60 years. AD has limited treatment options, and therefore efforts to prevent or delay its onset are the chief priority for ensuring the health of aging population (1, 2). The gradual, cumulative brain changes that lead to AD occur over several decades prior to the first signs of cognitive impairment and this provides a window of opportunity for disease-modifying/prevention interventions (3-6). Yet the life-long action of AD risks on an individual is not well-understood and this limits the efforts to evaluate personalized risk mitigation strategies that may delay or prevent AD (5). Existing approaches are focused on early diagnosis of people at risk for AD and use a combination of amyloid- or tau-sensitive positron emission tomography (PET), blood and cerebrospinal fluid (CSF) analysis, MRI-based brain morphometry, and neurobehavioral clinical/cognitive assessments (5). These approaches straddle sensitivity versus specificity versus cost, invasiveness, and availability concerns and are generally not suitable for capturing both effects of AD risk factors in typically aging samples and for predicting conversion to AD in high-risk individuals. Structural MRI comes close to this because it is a non-invasive and widely available alternative for assessing preclinical AD risk. However, clinical MRI-based findings in AD, such as hippocampal shrinkage and ventricular enlargement, are non-specific and typically emerge after the onset of prodromal symptoms (7-11).

To address these limitations, we propose an alternative strategy that uses structural MRI to capture regional similarities to AD-specific brain changes (i.e., brain deficit patterns)(12-14). We first conducted a meta-analysis of regional MRI effect sizes comparing amyloid PET-confirmed AD cases and amyloid-negative controls. From this, we ranked AD’s impact on brain structures and used this regional pattern to develop a Regional Vulnerability Index (RVI) for AD. RVI-AD is measuring the agreement between an individuals’ brain and the characteristic brain patterns in AD, rather than focusing solely on measuring individual brain structures such as the hippocampus or temporal cortex. Other multivariate approaches combine biomarkers, including amyloid pattern similarity scores (15), into a unifying measure using complex machine and deep learning methods (16-19). In contrast, RVI-AD is a linear measure applied to standard anatomical brain MRI. Here, we used RVI-AD to test the hypotheses that (1) known risk factors for AD act throughout adulthood, leading to the gradual formation of AD brain deficit patterns, and (2) the development of these patterns may predict dementia onset. If RVI-AD can capture these processes, it may allow for a mechanistic evaluation of how risk factors influence the brain’s structural progression from healthy aging to AD over time.

The first hypothesis was tested by tracking the actions of two established risk factors for AD in healthy adults. The apolipoprotein E (APOE) ε4 allele is the best-validated genetic risk factor for the late-onset form of AD, associated with a 3- to 15-fold elevation of the risk of developing AD (20-22) in people of central European ancestry (23). APOE is a cholesterol- and triglyceride-transporting lipoprotein with lipid binding sites (24) and has three major isoforms in humans (25). Both homozygous and heterozygous carriers of the ε4 isoform have a higher affinity for transporting low-density cholesterol and face a higher risk of developing AD with 30–60% developing dementia by age 85(26). We hypothesized that RVI-AD may capture brain abnormalities linked to AD (27-31) in the brains of healthy ε4 allele carriers.

Poor cardiovascular health is another significant risk factor and a key target for individualized efforts for dementia prevention (32-36). Conditions like coronary artery disease and myocardial infarction are associated with a 3- to 10-fold risk elevation for developing AD (37, 38). We quantified cardiovascular risks using the Framingham Cardiovascular Risk Score (FCVRS) (39). FCVRS is widely used to account for the effects of cholesterol, hypertension, and diabetes mellitus, in addition to age and sex, on future cardio- or cerebrovascular disease (39). FCVRS has been validated for predicting important health outcomes such as myocardial infarction, stroke, and death. The APOE ε4 allele and cardiovascular health are interlinked in AD-related pathogenesis; a higher affinity for low-density cholesterol transport leads to arterial stiffening, hypertension, and risks for neurological sequelae in middle age and beyond (40, 41), all of which are known risks for AD (36, 42).

We tested the first hypothesis by analyzing two cohorts of neurologically healthy, middle-aged to older adults at no immediate risk for AD. We tested if RVI-AD is sensitive to the action of known AD risk factors during neurologically normal state and compared it to effect sizes observed in different brain regions affected by AD, including hippocampus volume and frontal cortical gray matter thickness. Our discovery cohort consisted of Old Order Amish and Mennonite (OOA/M) participants, and the replication cohort consisted of UK Biobank (UKBB) participants. The OOA/M participants, of central European descent, share a rural, farm-based dwelling with low rates of alcohol, tobacco, and illicit substance use (43). This population has much better genetic, economic, and environmental uniformity compared to general populations. UKBB participants were recruited from urban and suburban populations. The vastly different environmental backgrounds of these two populations should provide a more rigorous replication test for the generalizability of the findings.

We tested the second hypothesis using longitudinal data from the Alzheimer’s Disease Neuroimaging Initiative (ADNI), which includes older age samples with higher risks for developing AD, to evaluate whether elevation of RVI-AD was associated with an increased risk of future dementia. An ideal early biomarker for AD should capture the risks associated with both APOE ε4 allele and cardiovascular health at a preclinical, healthy stage, as well as predict conversion to dementia when risk is elevated.

## Methods

### Risks at Normalcy: Discovery and Replication Samples

The discovery sample consisted of 343 OOA/M participants (140M/203F, age range 21–72 years, mean ± s.d.: 49.1 ± 12.7 years). Exclusion criteria included a history of epilepsy, cerebrovascular accidents, head injury with cognitive sequelae, intellectual disability, and unstable major medical conditions at the time of the study. Research participants with substance dependence within the past 6 months or current substance use disorder (except nicotine) were also excluded. Other exclusion criteria for this analysis included current or lifetime major psychiatric illness. Study participants gave written informed consent approved by the University of Maryland IRB.

The replication sample consisted of participants in the UK Biobank where we analyzed a subset of N=31,440 participants (mean age = 63.81 ± 7.44 years; 14,813M/16,627F) who were free of neuropsychiatric illnesses and for whom neuroimaging and clinical data were available. This included structural and diffusion white matter imaging phenotypes. Data were collected between 2012 and 2021 (36). The UK Biobank received ethics approval from the North West Multi-Center Research Ethics Committee (11/NW/03820).

### Imaging in ACP and UKBB

RVI were generated using structural and diffusion imaging data collected using similar imaging protocols and processing using consistent ENIGMA structural and diffusion pipelines, see Supplement.

#### Effect Sizes for Amyloid-Positive AD from ADNI and OASIS

NIA-AA has defined *biological* Alzheimer’s disease based on the presence of abnormal levels of beta-amyloid or tau protein in biofluids or on brain PET scans in subjects who experienced persistent clinical decline in one or more clinical domains, including memory (5). The regional vulnerability index for AD (RVI-AD) was calculated using the effect sizes derived between amyloid-positive (Aβ+) AD individuals and healthy amyloid-negative (Aβ−) stable controls from two datasets: the Alzheimer’s Disease Neuroimaging Initiative (ADNI) (132 Aβ + dementia cases and 223 Aβ− healthy controls) and the Open Access Series of Imaging Studies (OASIS) (36 Aβ + dementia cases and 180 healthy Aβ− controls). Altogether, imaging data for ADNI and OASIS datasets for 571 subjects–for whom Aβ status was available–served as the basis for the RVI-AD analysis.

We calculated Cohen’s *d* estimates for cortical thickness and subcortical gray matter differences region-by-region between 168 Aβ + dementia cases and 403 Aβ− healthy controls by combining the two datasets using ENIGMA structural imaging pipelines(43) (**Table S1**). These subjects lacked white matter diffusion imaging phenotypes, so white matter regional effect sizes were derived from a previous report on the ADNI participants. These effect sizes were calculated based on 48 AD cases and 53 controls also using the ENIGMA pipeline, although no specific Aβ status was considered there(44). In total, we extracted 64 regions from the whole brain, including 33 regional cortical thickness measures, 7 subcortical gray matter volumes and 24 regional white matter integrity measurements where data from left and right hemispheres were averaged (**Table S1**). To calculate Cohen’s *d*, we performed a linear regression for the diagnosis variable while including age and sex, their interaction, and the total intracranial volume as covariates, and performed *t*-to-*d* value conversion.

### ADNI participants and conversion to dementia

ADNI includes an ongoing, longitudinal multisite study of aging(45, 46). We tested how baseline RVI-AD values were associated with risks of conversion to dementia. Baseline neuroimaging summary data were downloaded from ADNI database (www.loni.usc.edu/ADNI) to create RVI-AD measurements for each participant. We also downloaded up to 12 years of follow up clinical data that documented whether the individual status has remained stable or changed to dementia or MCI. The final ADNI sample consisted of (N = 1,932, age = 73.6 ± 7.2, M/F = 1043/889) that included 586 cognitively normal (CN) older healthy controls (age = 73.8 ± 7.2, M/F = 286/318) and 965 participants with mild cognitive impairment (MCI, age = 72.9 ± 7.5, M/F = 569/397) at baseline. 380 participants with dementia at baseline (age = 75.1 ± 7.1, M/F/=207/173) were included as a comparison. These analyses excluded participants with Aβ status data available, as those data had been used to create effect sizes for RVI. Conversion information was not available for all participants because some had dropped out of the study or failed the follow up. For the available data in the CN at baseline group, N=35 (age = 75.2 ± 3.9. M/F = 17/18) had changed clinical status to dementia since baseline (average conversion period = 4.7 ± 2.5 years) and N=76 (average age = 75.5 ± 4.6, M/F = 41/49) had clinical status changed to MCI (average conversion period = 2.9 ± 2.6 years). In the MCI at baseline group, N=348 (age = 74.0 ± 7.0, M/F = 201/147) had clinical status changed to dementia since baseline (average conversion period = 2.3 ± 2.4 years).

#### Genotyping for APOE status.

All datasets provided APOE genotypes for all individuals and the genotyping methodology is provided in the Supplement.

### Framingham cardiovascular risk score

The Framingham cardiovascular risk score (FCVRS) is a weighted composite index developed to predict a 10-year risk of a major cardiovascular event such as heart attack, stroke or death(47). FCVRS scores were calculated for all subjects in the discovery and replication datasets using the uniform FCVRS algorithm(30). Only N=498 subjects in ADNI baseline sample had all of the measures needed to calculate FCVRS.

### Statistical analysis

#### RVI calculation

The Regional Vulnerability Index (RVI) scores were calculated using the ‘RVIpkg’ in [R] software based on our previous publication(48) with some revisions. The original RVI calculated the correlational agreement between an individual’s regional brain measures and the pattern of regional SSD-related brain deficits(49). It was used for patients with a diagnosis and required a sample of healthy controls to perform normalization [70]. It is therefore inappropriate for most of the current samples when participants at baseline had no AD. Accordingly, we computed the modified RVI as a normalized dot product of two vectors: the vector of individual regional deviations (coded as z-score deviations from the mean of the group) and the vector of AD effect sizes (Table S1). Specifically, the RVI was calculated for each subject as the dot product between vectors Z and E normalized by the dimensions of the vector using the following equation.

$$RVI={\sum \;}_{i=1}^{N}(Z_{i}\times E_{i})$$

where Z is the vector of deviation from the mean and E is the vector of meta-analytical effect size (Cohen’s d coefficients) for corresponding regional measures for AD. N is the dimension of the vector, i.e., the total number of imaging phenotypes for modalities including the whole-brain, cortical, subcortical, and white matter. Positive RVI values indicate that the regional pattern of an individual coincides with the expected pattern of AD. Therefore, the modified RVI version is similar to the original RVI in terms of relying on the regional effect sizes as the blueprint to define an individual’s similarity to the illness but using a full sample vector alignment rather than relying on healthy control group to achieve normalization.

#### Hypothesis testing

We first evaluated if individual RVI-AD derived from AD cases and Aβ− healthy controls can significantly identify the impact of the known genetic risk factor for AD - the APOE-ε4 genotype - on the AD-like brain patterns, using a Student’s *t*-test. This was followed by investigating the impacts of FCVRS and the joint APOE genotype and FCVRS effects on RVI-AD using linear regressions using Model 1:

(Model 1) $$\mathrm{RVI}\text{-}\mathrm{AD}∼\beta_{\varepsilon 4}\cdot E4+\beta_{\mathrm{FCVRS}}\cdot \mathrm{FCVRS}+\beta_{\varepsilon 4^{*}\mathrm{FCVRS}}\cdot E4^{*}\mathrm{FCVRS}$$

Primary analyses were performed on the whole brain RVI-AD with follow up analyses examining tissue-specific RVI-AD. We tested each hypothesis first in the ACP, and then sought replication in the UKBB samples.

Next, we used the ADNI dataset to test if higher RVI-AD values in people diagnosed as MCI are associated with risk for conversion to dementia. The primary analysis was in the MCI group, where we compared those who did not convert and remained stable (MCI→MCI) in the following decade to those who converted to dementia (MCI→dementia), using *t*-tests. We also compared these two groups to those with dementia at baseline and those who maintained the CN status.

We then also tested this in the CN group where a small cohort had converted to dementia, although the sample size for converters here was small: we compared those who did not convert and remained CN in the following decade (CN→CN) to those converted to dementia (CN→dementia) and also to those only converted to MCI but not dementia (CN→MCI) using t-tests.

To estimate the association between RVI-AD and the time to the onset of dementia, we further conducted logistic regression analysis to investigate the odds ratio (OR) of dementia conversion within MCI cohort (Model 2).


(Model 2) $$Conversion_{t}∼\beta_{RVI}RVI\text{-}AD+Age$$

Our analysis comprised of two parts. Firstly, we examined the odds ratio (OR) of conversion cases occurring annually. Second, we examined the OR of cumulative dementia conversion cases, over a 12-year follow-up period. For both components of the analysis, logistic regression models were fitted for each annual time point or by averaging of all 12 years of data, adjusting for baseline age.

Model 3 expanded the model 2 with two additional dementia risk factors (i.e., APOE-ε4 genotype and FCVRS scores) as predictors to assess if the joint models improved performance in explaining the variation in dementia conversion.


(Model 3) $$Conversion_{t}∼\beta_{RVI}RVI\text{-}AD+\beta_{\varepsilon 4}\cdot APOE\text{-}\varepsilon 4+\beta_{FCVRS}\cdot FCVRS\;+Age$$

Model performance was evaluated using likelihood ratio tests comparing the joint models to the null model. The OR of each predictor reflects the risk associated with a one-unit increase in the predictor. To be able to compare OR between predictors, the predictors were normalized. Note that we cannot directly model the “joint” OR of the predictors, but we can evaluate whether adding APOE4 and/or FCVRS improves the model fit using likelihood ratio tests, where a higher likelihood ratio value of a model indicates a better model fit. As only a portion of the ADNI participants have all the data needed to calculate FCVRS, we used only a subsample with all data available to test the full Model 3.

## Results

### APOE Genotype on RVI-AD in healthy samples: discovery and replication

#### Discovery sample

**Figure 1:**
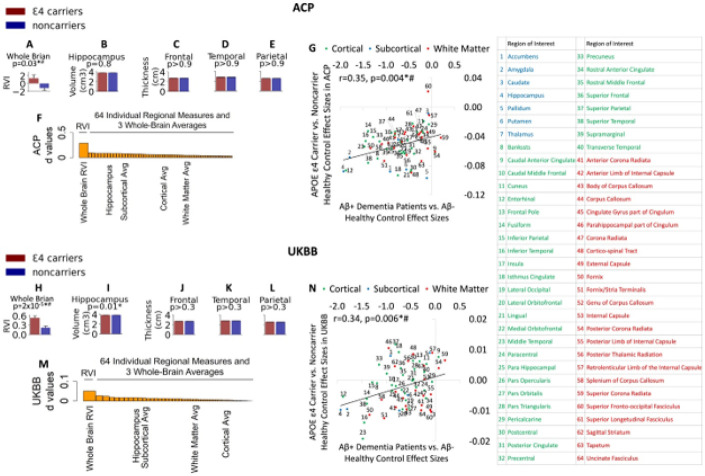
Healthy APOE4 carriers showed replicable Alzheimer’s disease (AD) brain patterns based on the whole-brain regional vulnerability index for AD (RVI-AD). **A, H**: RVI-AD in carriers was significantly higher than noncarriers in the Amish Connectome Project (ACP) discovery and the UK Biobank (UKBB) replication samples. Individual regional measures such as hippocampal volumes (**B, I**) and frontal, temporal, and parietal cortical thickness (**C-E, J-L**) showed weak and insignificant effects in both cohorts. The effect sizes for carriers vs. noncarriers were substantially larger in whole-brain RVI-AD compared to any individual regional measures including the averaging values of the cortical, subcortical, and white matter measures in both cohorts (**F, M**). **G, N**: Whole-brain RVI-AD was calculated by Alzheimer’s disease effect sizes of different regions (*x* axis), which showed significant correlation with effect sizes of the APOE genotypes in otherwise healthy people in both cohorts. *Statistically significant; # statistically significant difference was replicated across samples. Regions of interests are labeled in the table.

The N=91 APOE-ε4 carriers (38M/53F, age: 48.0 ± 12.5) exhibited significantly higher RVI-AD compared to non-carriers (Cohen’s *d* = 0.29, *p* = 0.03) (Fig. 1A). In comparison, the two groups had no significant differences in brain measurements in structures affected by AD, including hippocampal volume (d=−0.01, p = 0.8) and the cortical thickness of the temporal, parietal, and frontal cortical areas (all p > 0.7) (Fig. 1B-F). The effect sizes of APOE-ε4 on individual brain were significantly correlated with the pattern of effect sizes observed in AD (r = 0.35, p = 0.004, Fig. 1G).

#### UKBB sample

In the UKBB cohort, APOE-ε4 carriers demonstrated significantly higher RVI-AD compared to non-carriers (p = 2·10^−5^) (Fig. 1H). APOE-ε4 carriers had lower hippocampal volume (p = 0.01) but it was not significant after correction for multiple comparisons (Fig. 1I) and showed no other suggestively significant differences when compared to noncarriers (Fig. 1J-L). The APOE-ε4 effect sizes for individual brain region measurements were smaller than the RVI-AD effect size (Fig. 1M). The effect sizes of APOE-ε4 on individual brain regions in UKBB were also significantly associated with the AD effect sizes of these regions as identified by Aβ + AD patients (r = 0.34, p = 0.006, Fig. 1N).

### Effects of Cardiovascular Risk on RVI-AD

#### Discovery sample

**Figure 2:**
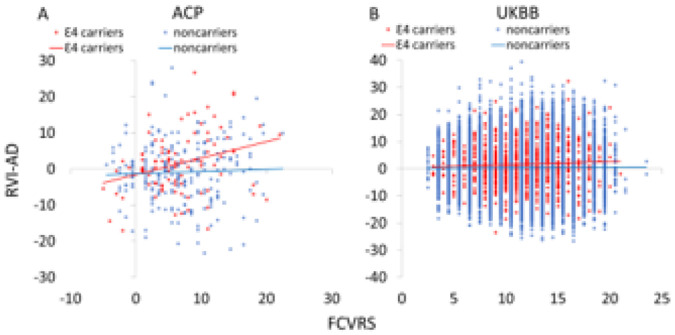
The Framingham cardiovascular risk score (FCVRS) were significantly associated with whole-brain regional vulnerability index for Alzheimer’s disease (RVI-AD) in APOE4 carriers in both the discovery (**A**) (left, r=0.35, p=4·10^−5^) and replication (**B**) samples (right, r=0.07, p=4·10^−7^). The correlation between RVI-AD and FCVRS in controls were r=0.02 and 0.0 in ACP and UKBB, respectively (p>0.9)

Model 1 was significant in the ACP cohort (F = 4.6, p = 0.0002, Table 1), with a significant main effect of FCVRS (p = 0.006) and a significant APOE · FCVRS interaction (p = 0.03, Table 1). This significant interaction was due to a stronger correlation between FCVRS and RVI-AD in APOE-ε4 carriers (r = 0.35, p = 4·10^−5^ vs. r = 0.02, p = 0.95) (Fig. 2A) and the difference in correlation coefficients was significant (z = 2.9, p = 0.004). Notably, the two genotype groups did not significantly differ in general cardiovascular risks as measured by FCVRS (6.5 ± 0.6 vs. 6.5 ± 0.4, p = 0.9), systolic, diastolic, or pulse pressure (117.4 ± 1.5, 72.0 ± 0.9 and 45.4 ± 1.3 vs. 117.2 ± 1.0, 72.6 ± 0.6 and 44.5 ± 0.7; all p > 0.4), suggesting that the APOE-ε4 allele by itself did not significant impact peripheral cardiovascular measures.

#### UKBB sample

Model 1 was significant in the UKBB cohort (F = 8.8, p = 7·10^−6^). We observed significant effects of FCVRS (p = 2·10^−4^), APOE-ε4 genotype (p = 0.01), and their interaction (p = 5·10^−4^) on RVI-AD (Table 1). The correlation between FCVRS and RVI-AD was significant in APOE-ε4 carriers (r = 0.07, p = 4·10^−7^) but not in noncarriers (r = 0.00, p = 0.7), and the difference in correlation coefficients was significant (z = 3.7, p = 2·10^−4^). (Fig. 2B). APOE-ε4 carriers and non-carriers did not differ in FVCRS (11.4 ± 0.02 vs. 11.3 ± 0.2, p = 0.07) or systolic, diastolic or pulse pressure (139.9 ± 0.7, 78.0 ± 0.4, and 61.8 ± 0.5 versus 140.9 ± 0.2, 78.7 ± 0.5, and 62.1 ± 0.3, respectively) (all p > 0.2). We also evaluated RVI-AD calculated separately for cortical thickness, subcortical gray matter volumes, and white matter and observed similar trends. Overall, whole-brain RVI-based models demonstrated the highest robustness and consistency in capturing the APOE and FCVRS risks (see **Table S2**).

### Predicting conversion from MCI to AD in ADNI participants

**Figure 3:**
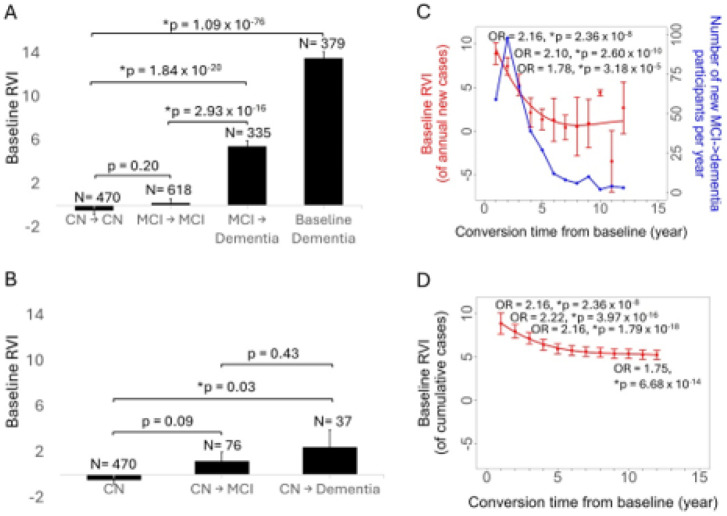
**A**. Baseline MCI participants’ whole-brain RVI-AD effects, where conversion (MCI→dementia) cases showed significantly higher baseline RVI-AD than no conversion (MCI→MCI), and importantly, MCI without conversion showed no significant difference to cognitively normal (CN) elderly healthy controls. The CN→CN group from **B** and the dementia cases already diagnosed at baseline are included here for easier comparison. **B**. Baseline cognitive normal (CN) participants’ whole-brain RVI-AD effects. CN→CN: CN individuals at baseline who remained CN during the 12-year follow-up; CN→MCI: those converted to MCI; CN→dementia: converted to dementia. **C**. Mean and s.e. of baseline RVI of annual new AD cases in the MCI participants: high baseline RVI had a significantly higher odds ratio (OR) for predicting conversion from MCI to dementia in the next three years after the baseline MRI. **D**. Mean and s.e. of baseline RVI of cumulative cases: high baseline RVI had a significantly higher odds ratio (OR) for predicting conversion from MCI to dementia in each of the 12 year follow-up.

We used logistic regression to evaluate the odds ratio (OR) of conversion to dementia (see Models 2 in Methods) in ADNI participants. Out of N=965 participants with MCI, N=335 (age = 74.0 ± 7.0, 201 M/147 F) developed dementia over the next 12 years, with an average conversion period of about 3 years. Participants who converted to dementia had significantly higher baseline RVI compared to those who did not (p = 3·10^−16^) (Table 2, Fig. 3A). MCI individuals who did not convert to dementia in a decade showed no significant difference in RVI from cognitively normal (CN) elderly adults who remained CN 12 years later (Fig. 3A).

In addition, N=37 out of the M = 586 cognitively normal ADNI participants (age = 75.2 ± 3.9. M/F = 17/18) also developed dementia (CN→dementia) after an average conversion period of ~ 5 years. This small sample also showed significantly higher baseline RVI-AD compared to CN participants who remained stable (CN→CN) (p = 0.03) (Fig. 3B). In comparison, the N=76 CN participants who developed MCI during the same period (CN→MCI) had intermediate RVI values between the CN→dementia and CN→CN (N = 461) groups.

We used an annual logistic regression analysis to test the timing of the odds ratio for predicting new conversions to dementia based on RVI-AD. Higher baseline RVI-AD was significantly associated with conversion from MCI to annual new dementia cases in each of the first three years (OR = 2.2 to 1.78, p = 3·10^−5^ to 3·10^−10^) (Fig. 3C). In aggregate, the odds ratio for the 12-year cumulative conversion to dementia was highly significant (OR = 1.8, p = 7·10^−14^) as shown by the last data point in Fig. 3D (which also plotted the OR of annual cumulative conversion).

We expanded the logistic regression model to include APOE-ε4 genotype status and FCVRS as additional predictors (see Model 3 in Methods). This analysis was conducted on N=498 participants with available FCVRS data, out of whom N=243 MCI participants converted to dementia. Both APOE-ε4 genotype (OR = 1.2, p = 0.01) and RVI-AD (OR = 1.7, p = 5·10^−5^) contributed to dementia conversion over the 12-year follow-up period. FCVRS did not contribute significantly to dementia conversion (OR = 0.0). This suggests that in this age range and high-risk stage of MCI, FCVRS effects for conversion may no longer be prominent.

We evaluated the area under the curve (AUC) for predicting conversion from MCI to dementia and found that the peak AUC was 74%. Including the APOE genotype only slightly improved the AUC to 75% (Table S3, see supplement). In comparison, the model that only had FCVRS + APOE4 status showed AUC of 0.57.

The predictive ability of baseline RVI-AD values for conversion to dementia declined with time (Fig. 3C). To estimate RVI-AD’s long-term predictive value as part of a hypothetical regular screening program, we simulated annual, triennial, and quinquennial structural MRI assessments (Methods in **Supplementary Information**). The simulations suggest that once MCI is diagnosed, RVI-AD calculated based on annual MRI had the strongest performance (AUC = 0.80), and even triannual follow-up still had clinically relevant AUC (AUC = 0.75) for a ten-year prediction of conversion to dementia (**Figure S1**).

## Discussion

We evaluated the regional vulnerability index for Alzheimer’s Disease (RVI-AD) to gauge its ability to track the lifelong contributions of established risk factors that eventually culminate in dementia. RVI-AD quantifies an individual’s similarity to AD brain deficit patterns. We used a meta-analytical sample of AD patients with amyloid positive (Aβ+) status and amyloid negative controls to estimate this pattern. In healthy adults, RVI-AD showed a positive correlation with the subtle actions of known APOE-ε4 genetic effects as well as the cardiovascular risks, as measured by Framingham Cardiovascular Risk Score (FCVRS). We observed a significant additive ε4-by-FCVRS effect on RVI-AD, indicating that comparable levels of cardiovascular risks led to a higher similarity to AD patterns in people with the APOE-ε4 genotype. With a few exceptions, these findings were consistent in both farm-dwelling Amish and in urban and suburban UK participants. Higher RVI-AD in the participants ascertained by Alzheimer’s Disease Neuroimaging Initiative (ADNI) was significantly associated with the elevated risk for conversion to dementia. Simulations suggested that RVI-AD could support a screening program that uses annual-to-triannual structural MRI scans to provide clinically relevant (AUC = 0.75–0.80) assessment of risk for conversion to dementia in people diagnosed with MCI. RVI-AD is a novel and promising biomarker capable of capturing both the preclinical AD risk factors as well as predicting the likelihood of dementia development in individuals with MCI.

The presence of illness-like brain patterns reflects the cumulative action of associated risk factors and may be informative of the risk of developing a disorder (50). We tested this hypothesis using two best-known AD risks: APOE-ε4 genotype and cardiovascular factors. The effects of the APOE-ε4 allele on elevating RVI-AD were stable and replicable in two cohorts where the APOE-ε4’s effects on RVI-AD elevation acted primarily through cardiovascular risks. APOE-ε4 is a significant risk factor for cardio-and-cerebrovascular conditions (27, 57-62). The burden of cardiovascular factors contributes up to 50% of the risk for developing of late-onset AD(51, 63, 64) and while there were no significant differences in FCVRS or blood pressure measures in APOE-ε4 carriers vs. non-carriers, RVI-AD still detected the additional brain effects linked to cardiovascular risks in the carriers. Incremental elevations in FCVRS in APOE-ε4 carriers resulted in a greater resemblance to AD-like brain patterns compared to non-carriers, suggesting that cardiovascular risk factors may act on the brain differently in APOE- ε4 carriers. Thus, RVI may provide an early biomarker to index this genetic + cardiovascular risk effect for developing an AD-like brain pattern at the level of the individual at cognitive and medical normalcy.

We tested the second hypothesis in individuals with MCI (mean age ~ 74 years) who were at risk for AD because approximately one third of subjects went on to develop dementia (6, 65). MCI subjects who developed dementia had significantly higher baseline RVI-AD indices compared to participants who remained stable. This finding was also replicated in the cognitively normal controls. The predictive accuracy of conversion was strongest within three years after the MRI scan. This suggested that additional neurodegeneration needs to occur for MCI to dementia conversion and that annual-to-triannual MRI assessments in people at risk for dementia could maintain clinically acceptable AUC. A structured RVI-based screening program could supplement or provide a cost-effective alternative to AD-focused PET/CSF assessments. Importantly, MCI participants who did not progress to dementia had RVI-AD values similar to the RVI values for the cognitively normal controls (Fig. 3A), suggesting that low RVI-AD predicted reduced likelihood for conversion. Approximately half of the MCI individuals never develop dementia (6, 66, 67), leading to a decision challenge for disease-modifying treatments that have significant side effects. Present approaches to evaluate individual’s risks, include tau-focused PET scans, volumetric MRI assessments, cognitive batteries, and blood/CSF biomarkers (11, 68-70). Many of these approaches have high costs and perceived invasiveness (73). In contrast, RVI-AD uses linear, easily interpretable calculations and measurements from readily available structural MRI. Further validation may enable RVI-AD index to assist in early risk detection and predict more imminent dementia disease onset, especially when combined with other available clinical and biomarker measures.

In conclusion, RVI-AD can detect the early, lifetime gradual risk factor effects on the brain before cognitive changes emerge, as well as the more imminent risks for conversion of MCI to AD. AD is a major cause of cognitive dysfunction among older people. With new AD treatments on the horizon, non-invasive brain imaging biomarkers, such as RVI-AD, could identify vulnerable individuals during cognitively normal to prodromal stages, supporting public health efforts to prevent, delay, or reduce the impact of AD.

## Supplementary Material

This is a list of supplementary files associated with this preprint. Click to download.

• Supplement1.docx

## Figures and Tables

**Table 1:** Evaluating Model 1 in participants of Amish Connectome Project (ACP) and UK biobank (UKBB) studies. Values are mean ± SEM of the beta values (is this correct???)

Sample	Model Statistics	APOE4	FCVRS	APOE4 x FCVRS
ACP (N = 343)				
Whole-Brain RVI-AD Value	F = 4.6, p = 0.002	−0.14 ± 0.9 (t=−0.1,p = 0.9)	0.25 ± 0.09 (t = 2.8,p = 0.006)	0.20 ± 0.09 (t = 2.1,p = 0.03)
UKBB (N = 31,440)				
Whole-Brain RVI-AD Value	F = 8.8, p = 7·10^−6^	0.95 ± 0.4 (t = 2.3,p = 0.01)	0.13 ± 0.03 (t = 3.7,p = 2·10^−4^)	0.12 ± 0.03 (t = 3.5,p = 5·10^−4^)

**Table 2:** Evaluating Models 2 and 3 in the odds ratio (OR) for predicting conversion from MCI to dementia. The values are OR, 95% confidence interval and p-values for MCI subjects whose clinical status changed to dementia over the assessment period.

MCI→AD *Conversion Models*	*Age*	RVI-AD	*APOE4*	*FCVRS*
Model 2 (N = 965) Odds Ratio	1.02 (1.01–1.05), p = 0.005	1.74 (1.52–2.03), p = 2·10^−5^		
Model 3 (N = 498) Odds Ratio	1.03 (0.99–1.07), p = 0.2	1.35 (1.10–1.64), p = 0.004	1.24 (1.04–1.4), p = 0.01	1.08 (0.90–1.3), p = 0.9

## Significance

Alzheimer’s disease (AD) is the leading cause of cognitive and functional aging-related disability and has limited treatment options. Efforts to prevent or delay its onset are the chief priority for ensuring the health of an aging population. This requires sensitive and specific biomarkers capable of tracking the action of AD risk factors at the stage of clinical normalcy. We developed a Regional Vulnerability Index (RVI) that quantifies individual brain similarity to AD patients' expected brain deficit patterns. We tested two overall hypotheses that RVI-AD is sensitive to the best-known risk factors for AD in young-to-middle-aged individuals (APOE-e4 genotype and cardiovascular risks) and that elevation in RVI-AD can predict conversion to dementia in older individuals with mild cognitive impairment (MCI).
